# Total Synthesis of Gobiusxanthin Stereoisomers and Their Application to Determination of Absolute Configurations of Natural Products: Revision of Reported Absolute Configuration of Epigobiusxanthin

**DOI:** 10.3390/md13010159

**Published:** 2014-12-30

**Authors:** Yumiko Yamano, Kotaro Ematsu, Hiromasa Kurimoto, Takashi Maoka, Akimori Wada

**Affiliations:** 1 Department of Organic Chemistry for Life Science, Kobe Pharmaceutical University, Motoyamakita-machi, Higashinada-ku, Kobe 658-8558, Japan; E-Mails: hm090930@st.kobepharma-u.ac.jp (H.K.); a-wada@kobepharma-u.ac.jp (A.W.); 2 Research Institute for Production Development, 15 Shimogamo-morimoto-cho, Sakyo-ku, Kyoto 606-0805, Japan; E-Mail: maoka@mbox.kyoto-inet.or.jp

**Keywords:** carotenoid, gobiusxanthin, epigobiusxanthin, total synthesis, chiral HPLC separation, absolute configuration

## Abstract

(3*R*)-Gobiusxanthin stereoisomers (**1a**–**d**) were synthesized by stereoselective Wittig reaction of the (3*R*)-C_15_-acetylenic tri-*n*-butylphosphonium salt **7** with C_25_-apocarotenal stereoisomers **5a**,**b** and **14a**,**b** bearing four kinds of 3,6-dihydroxy-ε-end groups. The validity of the reported stereochemistry of gobiusxanthin was demonstrated by the fact that the reported spectral data of natural gobiusxanthin were in agreement with those of synthetic (3*R*,3'*S*,6'*R*)-gobiusxanthin (**1a**). On the other hand, the reported CD spectral data of natural epigobiusxanthin, which has been assigned as (3*R*,3'*R*,6'*R*)-isomer (3'-epigobiusxanthin), were identical with those of synthetic (3*R*,3'*S*,6'*S*)-isomer **1d** (6'-epigobiusxanthin) rather than those of the corresponding synthetic 3'-epi-isomer **1b**. It was found that the stereochemistry at C3-position has little effect on the shape of their CD spectra. Thus, in order to reinforce the validity of the absolute configurations at C3-position of natural specimens, (3*S*,3'*S*,6'*R*)- and (3*S*,3'*S*,6'*S*)-stereoisomers **1e** and **1f** were also synthesized and a HPLC analytical method for four stereoisomers was established by using a column carrying a chiral stationary phase. The HPLC analysis has proven that the stereochemistry of the natural epigobiusxanthin is 3*R*,3'*S*,6'*S*.

## 1. Introduction

**Figure 1:**
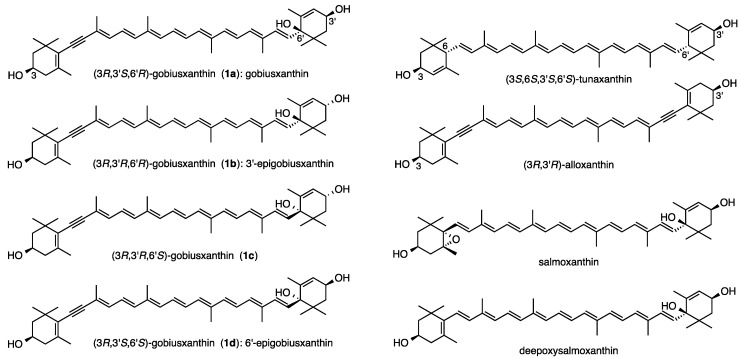

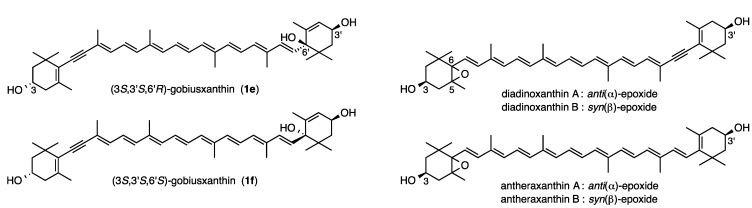
Structures of stereoisomers of gobiusxanthin (**1a**–**f**) and other related carotenoids.

Gobiusxanthin (**1a**) (Figure 1), which bears a novel 3',6'-dihydroxy-ε-end group, was first isolated from the common freshwater goby *Rhinogobius brunneus* [1] and then from the salmon *Oncorhynchus keta* [2]. Its structure was determined to be 7,8-didehydro-β,ε-carotene-3,3',6'-triol by MS and ^1^H-NMR spectroscopies and the absolute configuration was tentatively assigned as 3*R*,3'*S*,6'*R* from the resemblance of its CD spectrum to the calculated one of half (3*S*,6*S*,3'*S*,6'*S*)-tunaxanthin and half (3*R*,3'*R*)-alloxanthin according to the additivity rule of CD spectra [3]. From the salmon* Oncorhynchus keta*, salmoxanthin and deepoxysalmoxanthin, possessing the same 3',6'-dihydroxy-ε-end group, were also isolated together with gobiusxanthin [2]. Their absolute configurations were similarly postulated by comparing their CD spectra with those of analogous compounds. Recently, we accomplished the first total synthesis of these two carotenoids and consequently confirmed that their proposed configurations are correct [4]. The stereoisomer of gobiusxanthin, 3'-epigobiusxanthin (**1b**) was isolated from the crown-of-thorns starfish *Acanthaster planci* [5]. Its *trans*-configuration of the two hydroxy groups at C3' and C6' was determined by NOESY experiment and a 6'*R* configuration was estimated from the fact that it showed the negative Cotton effect around 280 nm in the CD spectrum [3]. In order to obtain an additional proof on the stereochemistries of gobiusxanthin (**1a**) and 3'-epigobiusxanthin (**1b**), we expected that efficient combination of a sterically-defined synthesis of authentic stereoisomers, spectroscopic analyses including NMR and CD, and a HPLC separation using a chiral column could be beneficial.

## 2. Results and Discussion

### 2.1. Synthesis of Gobiusxanthin (**1a**) and 3'-Epigobiusxanthin (**1b**)

**Scheme 1:**
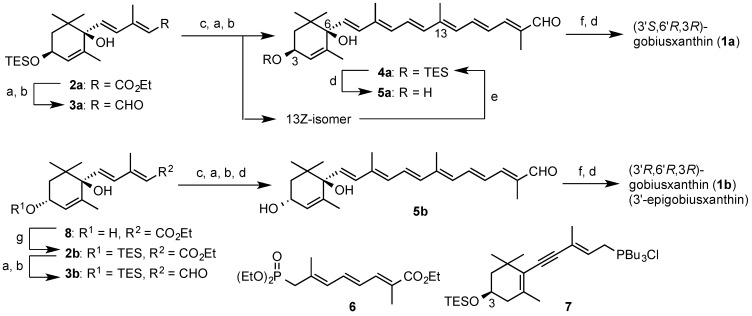
Synthesis of gobiusxanthin (**1a**) and 3'-epigobiusxanthin (**1b**). *Reagents*: (**a**) LAH; (**b**) MnO_2_; (**c**) phosphonate **6**, *n*-BuLi, *N*,*N*'-dimethylpropyleneurea (DMPU); (**d**) TBAF-AcOH; (**e**) PdCl_2_(MeCN)_2_, Et_3_N; (**f**) phosphonium salt **7**, NaOMe; (**g**) TESCl.

We previously reported [6] that the C_15_-acetylenic tri-*n*-butylphophonium salt **7** (Scheme 1) is a useful tool for stereoselective synthesis of acetylenic carotenoids. In addition, the triethylsilyl (TES)-protected 3,6-*syn*-dihydroxydienoate **2a** and the 3,6-*anti*-dihydroxydienoate **8** have already been prepared [4] in the course of synthesis of salmoxanthin. Thus, we synthesized gobiusxanthin (**1a**) and 3'-epigobiusxanthin (**1b**) by stereoselective Wittig reaction of the C_15_-phosphonium salt **7** with C_25_-apocarotenals **5a** and **5b**, which were derived from compounds **2a** and **8**, as shown in Scheme 1.

The C_15_-*syn*-dihydroxydienoate **2a** [4] was subjected to lithium aluminum hydride (LAH) reduction and subsequent MnO_2_ oxidation to afford the dienal **3a** in 61% yield. This was then condensed with the previously reported C_10_-phosphonate **6** [7], and the resulting hexaenoate was reduced with LAH and followed by MnO_2_ oxidation to provide TES-protected all-*E*-apocarotenal **4a** and its 13*Z*-isomer in 46% and 16% yield from **3a**, respectively. The 13*Z*-isomer was converted (52%) into the desired all-*E*-isomer by isomerization using a palladium catalyst [4,8]. After treatment of compound **4a** with tetrabutylammonium fluoride (TBAF), the resulting deprotected apocarotenal **5b** was condensed with phosphonium salt **7** under previously reported [6] conditions (NaOMe in CH_2_Cl_2_) and then desilytated to provide gobiusxanthin in 69% over the 3 steps.

After protection (quant.) of C_15_-*anti*-dihydroxydienoate **8** [4], the resulting TES-ether **2b** was transformed into 3'-epigobiusxanthin (**1b**) via the stereoselective condensation of C_25_-apocarotenal **5b** with the C_15_-acetylenic tri-*n*-butylphophonium salt **7**, in the same procedure as synthesis of gobiusxanthin (**1a**).

**Figure 2:**
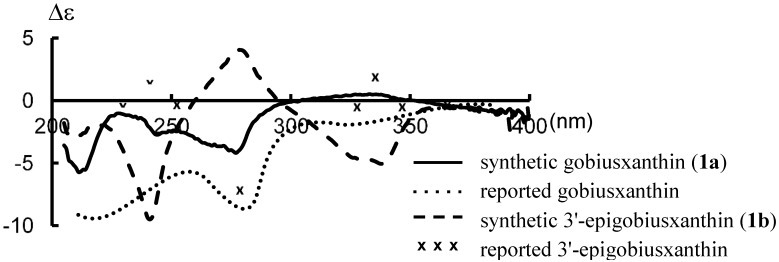
CD spectra in Et_2_O-isopentane-EtOH (5:5:2) of synthetic gobiusxanthin (**1a**), 3'-epigobiusxanthin (**1b**) and their reported spectra.

^1^H-NMR spectral data of synthetic **1a** and ^1^H- and ^13^C-NMR spectral data of synthetic **1b** were in good agreement with those of the reported data of goboiusxanthin [1] and 3'-epigobiusxanthin [5], respectively. As shown in Figure 2, the CD spectrum of synthetic **1a** was basically identical with the reported spectrum of gobiusxanthin, except for the intensities. On the other hand, the CD spectrum of the synthetic **1b** was opposite in sign to that of the reported 3'-epigobiusxanthin, indicating the reported absolute stereochemistry needs a correction. To make sure the stereochemistry of natural epigobiusxanthin, (6'*S*)-stereoisomer was also prepared as a reference sample.

### 2.2. Synthesis of Gobiusxanthin Stereoisomers **1c**–**1f**

**Scheme 2:**
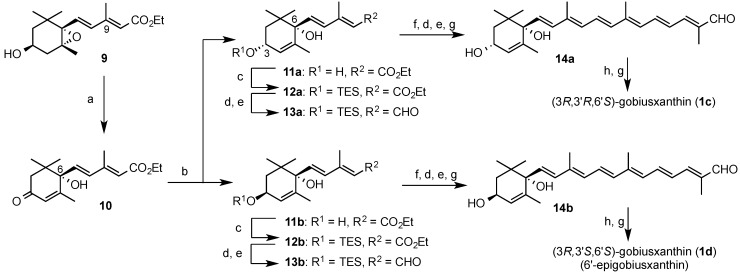
Synthesis of (6'*S*)-gobiusxanthin stereoisomers **1c** and **1d**. *Reagents*: (**a**) DMP then SiO_2_; (**b**) 9-BBN; (**c**) TESCl; (**d**) LAH; (**e**) MnO_2_; (**f**) phosphonate **6**, *n*-BuLi, DMPU; (**g**) TBAF-AcOH; (**h**) phosphonium salt **7**, NaOMe.

According to the procedure for the preparation of the corresponding enantiomer **8** [4], (3*S*,6*S*)-*anti*-diol **11b** was prepared as shown in Scheme 2. Previously prepared *anti*(α)-epoxide **9** [9] was oxidized with Dess-Martin periodinane (DMP) and then treated with a large amount of silica gel in AcOEt to afford (6*S*)-hydroxyenone **10** in 82% yield. Reduction of **10** with 9-borabicyclo[3.3.1]nonane (9-BBN) [4,10] gave (3*S*,6*S*)-*anti*-diol **11b** (44%), together with (3*R*,6*S*)-*syn*-diol **11a** (47%). *Anti*-diol **11b** was converted into (3*R*,3'*S*,6'*S*)-gobiusxanthin (**1d**) in the same procedure as synthesis of **1a** and **1b**. (3*R*,3'*R*,6'*S*)-Gobiusxanthin (**1c**) was also synthesized from *syn*-diol **11a**.

**Figure 3:**
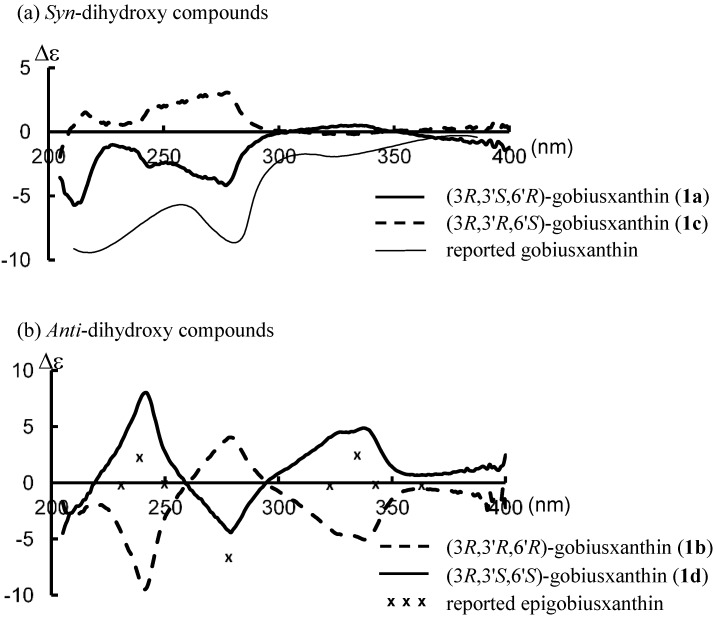
CD spectra in Et_2_O-isopentane-EtOH (5:5:2) of (3*R*)-gobiusxanthin stereoisomers (**1a**–**d**) and reported spectra of gobiusxanthin and 3'-epigobiusxanthin.

As shown in Figure 3b, the reported CD spectral data [5] of natural epigobiusxanthin shown by “x” were basically identical in the shape with those of synthetic (3*R*,3'*S*,6'*S*)-isomer **1d** (6'-epigobiusxanthin). It was found that the stereochemistry at C3-position has little effect on the shape of their CD spectra; CD spectrum of (3*R*,3'*S*,6'*R*)-gobiusxanthin (**1a**) showed an antisymmetrical shape to that of (3*R*,3'*R*,6'*S*)-gobiusxanthin (**1c**) (Figure 3a), whereas that of (3*R*,3'*R*,6'*R*)-gobiusxanthin (**1b**) showed an antisymmetrical shape to that of (3*R*,3'*S*,6'*S*)-gobiusxanthin (**1d**) (Figure 3b). The stereochemistry at C3'-position has a decisive influence on the shape and the sign of their CD spectra. It indicates that the C3-absolute configurations of gobiusxanthin and epigobiusxanthin cannot be determined exactly by CD spectra.

**Figure 4:**
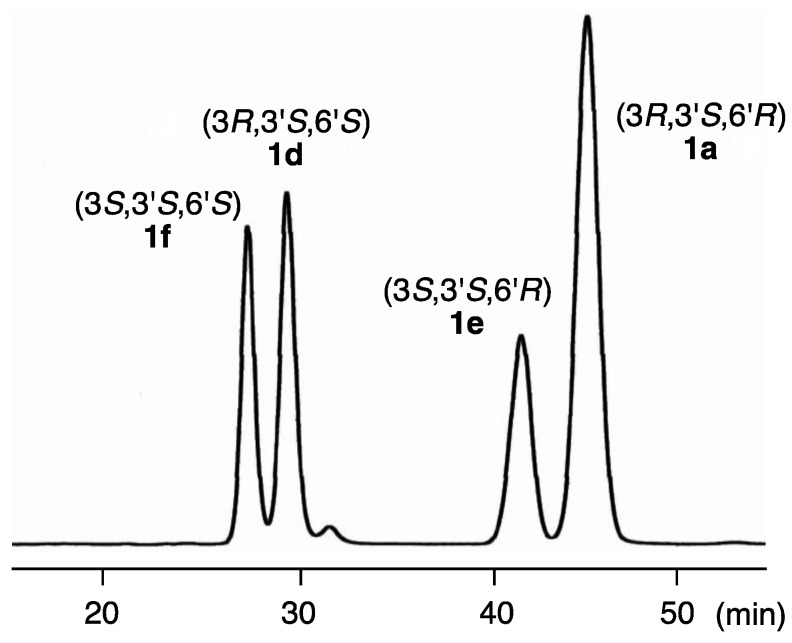
HPLC elution profile of a mixture of four stereoisomers of gobiusxanthin. Column: CHIRALPAK IB 0.46 × 25 cm; eluent: EtOH–*n*-hexane–CH_2_Cl_2_ (1:75:25); flow rate: 1.0 mL/min; temperature: 23 °C; detection: 450 nm.

Previously, we reported [11] that most alloxanthin specimens (Figure 1) isolated from various aquatic animals consist of only (3*R*,3'*R*)-stereoisomer; however, those from some aquatic animals are exceptionally mixtures of three stereoisomers. Thus, in order to reinforce the validity of the absolute configurations at C3-position of natural specimens, (3*S*,3'*S*,6'*R*)- and (3*S*,3'*S*,6'*S*)-stereoisomers **1e** and **1f** were also synthesized by Wittig condensation of C_25_-apocarotenals **5a** and **14b** with the enantiomers [11] of C_15_-phosphonium salt **7** and a HPLC analytical method for four stereoisomers **1a**, **1d**, **1e** and **1f** was investigated. As a result, these stereoisomers can be separated using a chiral column (CHIRALPAK IB; Daicel, Tokyo, Japan) as shown in Figure 4.

Application of this method to epigobiusxanthin isolated from the crown-of-thorns starfish *Acanthaster planci* [5] demonstrated that its stereochemistry is 3*R*,3'*S*,6'*S*. As described above, the absolute stereochemistries at C3'- and C6'-positions of gobiusxanthin were successfully to be 3'*S* and 6'*R* based of their identical CD spectra. Once we get gobiusxanthin specimen from a natural source, the absolute stereochemistry of C3-position can be unambiguously determined by the HPLC analysis.

The *syn*-configuration of the hydroxyl group at C3 and the epoxide oxygen was firstly identified in carotenoids, diadinoxanthin B and antheraxanthin B (Figure 1), isolated from the common freshwater goby *Rhinogobius brunneus* [1]. Diadinoxanthin B would be involved in a biosynthetic pathway of gobiusxanthin. On the contrary, epigobiusxanthin, which was found to be 6'-epi-form rather than 3'-epi-form, would be derived from common *anti*(α)-epoxy carotenoids such as diadinoxanthin A.

## 3. Experimental Section

### 3.1. General

UV-VIS spectra were recorded on a JASCO V-650 instrument (JASCO, Tokyo, Japan), with ethanol solutions. IR spectra were measured on a Perkin-Elmer spectrum 100 FT-IR spectrometer (Perkin-Elmer, Yokohama, Japan), with chloroform solutions. ^1^H- and ^13^C-NMR spectra were determined on a Varian Gemini-300 or a VXR-500 superconducting FT-NMR spectrometer (Agilent Technologies, Santa Clara, CA, USA), with deuteriochloroform solutions (tetramethylsilane as the internal reference). *J* Values are given in Hz. Mass spectra were taken on a Thermo Fisher Scientific Exactive spectrometer (Thermo Fisher Scientific, Bremen, Germany). CD spectra were measured on a Shimadzu-AVIN 62A DS circular dichroism spectrometer (Shimadzu, Kyoto, Japan). The concentrations were calculated using log ε = 5.0 at main λ_max_ (in EPA). Optical rotations were measured on a JASCO P-2200 polarimeter (JASCO, Tokyo, Japan).

HPLC analyses were performed on Simadzu-LC-20AT instrument (Shimadzu, Kyoto, Japan) with a photodiode array detector (Waters 996, Tokyo, Japan) and column oven (GL Sciences Model 552, Tokyo, Japan). Flash column chromatography (CC) was performed on using Kanto Silica Gel 60 N. Preparative HPLC was carried out on a Shimadzu LC-6A with a UV-VIS detector (Shimadzu, Kyoto, Japan).

All operations were carried out under nitrogen or argon. Evaporation of the extract or the filtrate was carried out under reduced pressure. Ether refers to diethyl ether, and hexane to *n*-hexane. NMR assignments are given using the carotenoid numbering system.

### 3.2. Synthesis of Gobiusxanthin (**1a**) and 3'-Epigobiusxanthin (**1b**)

(2*E*,4*E*)-5-[(1*R*,4*S*)-1-Hydroxy-2,6,6-trimethyl-4-triethylsilyloxycyclohex-2-en-1-yl]-3-methyl-penta-2,4-dienal (**3a**). A solution of ester **2a** [4] (1.27 g, 3.11 mmol) in dry ether (15 mL) was added dropwise to a stirred suspension of LAH (118 mg, 3.11 mmol) in dry ether (20 mL) at 0 °C. After being stirred at 0 °C for 10 min, the excess of LAH was decomposed by dropwise addition of moist ether and the mixture was filtered through a pad of Celite. The filtrate was dried and evaporated to give the crude alcohol, which without purification, was dissolved in ether (15 mL) and hexane (15 mL) and stirred with MnO_2_ (7.5 g) at rt for 30 min. After MnO_2_ was filtered off, the filtrate was concentrated. The resulting residue was purified by flash CC (AcOEt-hexane, 3:7) to afford the aldehyde **3a** (697 mg, 61% from **2a**): [α]_D_^22^ −149.1 (*c* 1.26, MeOH); IR ν 3600, 3447 (OH), 1661 (conj. CO), 1631, 1600 (C=C); ^1^H-NMR (300 MHz) *δ* 0.63 (6H, q,* J* 8, SiC*H*_2_ × 3), 0.95, 0.97 (each 3H, s, *gem*-Me), 0.98 (9H, t, *J* 8, CH_2_*Me* × 3), 1.64 (3H, t, *J* 1.5, 5-Me), 1.68 (2H, m, 2-H_2_), 2.27 (3H, d, *J* 1, 9-Me), 4.26 (1H, m, 3-H), 5.56 (1H, br s, 4-H), 6.01 (1H, br d, *J* 8, 10-H), 6.17 (1H, d, *J* 16, 7-H), 6.56 (1H, d, *J* 16, 8-H), 10.12 (1H, d, *J* 8, CHO); ^13^C-NMR (75 MHz) *δ* 4.79 (C × 3), 6.80 (C × 3), 13.32, 19.26, 24.38, 24.46, 38.52, 42.60, 65.29, 77.39, 129.18, 129.43, 133.29, 136.77, 138.82, 153.68, 191.49; HRMS (ESI) *m*/*z* calcd for C_21_H_36_O_3_NaSi (M + Na)^+^ 387.2326, found 387.2317.

(2*E*,4*E*,6*E*,8*E*,10*E*,12*E*)-13-[(1*R*,4*S*)-1-Hydroxy-2,6,6-trimethyl-4-triethylsilyloxycyclohex-2-en-1-yl]-2,7,11-trimethyltrideca-2,4,6,8,10,12-hexaenal (**4a**). To a solution of C_10_-phosphonate **6** [7] (1.90 g, 5.76 mmol) and *N*,*N*'-dimethylpropyleneurea (DMPU) (0.69 mL, 5.76 mmol) in dry THF (20 mL) was added *n*-BuLi (1.63 M in hexane; 3.64 mL, 5.94 mmol) at −20 °C. After being stirred at −20 °C for 15 min, a solution of the aldehyde **3a** (700 mg, 1.92 mmol) in dry THF (10 mL) was added to the reaction mixture and stirring was continued at −20 °C for 20 min. After being quenched with saturated aq. NH_4_Cl, the mixture was extracted with AcOEt. The extracts were washed with brine, dried and evaporated to give a residue, which was purified by flash CC (ether-hexane, 3:7) to provide an isomeric mixture of the hexaenoate (786 mg, 76%) as an yellow oil: IR ν 3599, 3518 (OH), 1692 (conj. CO), 1614, 1602, 1555 (C=C); HRMS (ESI) *m*/*z* calcd for C_33_H_52_O_4_NaSi (M + Na)^+^ 563.3527, found 353.3516.

A solution of this isomeric mixture in dry ether (20 mL) was added dropwise to a stirred suspension of LAH (83 mg, 2.2 mmol) in dry ether (20 mL) at 0 °C. After being stirred at 0 °C for 15 min, the excess of LAH was decomposed by dropwise addition of moist ether and the mixture was filtered through a pad of Celite. The filtrate was dried and evaporated to give the crude diol, which without purification, was dissolved in THF (1 mL), ether (15 mL) and hexane (15 mL), and then stirred with MnO_2_ (5.3 g) at room temperature (rt) for 30 min. After MnO_2_ was filtered off, the filtrate was concentrated. The resulting residue was purified by flash CC (AcOEt-hexane, 3:7) and then preparative HPLC [LiChrosorb Si 60 (7 μm) 2 × 25 cm; ether-hexane, 27:73] to afford all-*E*-apocarotenal **4a** (434 mg, 46% from **3a**) and its 13*Z*-isomer (149 mg, 16% from **3a**), as an orange foam, respectively.

all-*E*-Isomer **4a**: UV-VIS λ 421; IR ν 3598, 3521 (OH), 1661 (conj. CO), 1611, 1601, 1550 (C=C); ^1^H-NMR (300 MHz) *δ* 0.63 (6H, q,* J* 8, SiC*H*_2_ × 3), 0.94, 0.98 (each 3H, s, *gem*-Me), 0.98 (9H, t, *J* 8, CH_2_*Me* × 3), 1.65 (3H, t, *J* 1.5, 5-Me), 1.68 (2H, m, 2-H_2_), 1.88, 1.94, 2.04 (each 3H, s, 9-Me, 13-Me, 13'-Me), 4.25 (1H, m, 3-H), 5.53 (1H, m, 4-H), 5.67 (1H, d, *J* 15.5, 7-H), 6.23 (1H, br d, *J* 11.5, 10-H), 6.31 (1H, br d, *J* 12, 14-H), 6.38 (1H, d, *J* 15, 12-H), 6.45 (1H, d, *J* 15.5, 8-H), 6.68 (1H, dd, *J* 12, 14.5, 15'-H), 6.77 (1H, dd, *J* 11.5, 15, 11-H), 6.96 (1H, br d, *J* 12, 14'-H), 7.03 (1H, dd, *J* 12, 14.5, 15-H), 9.45 (1H, s, CHO); ^13^C-NMR (75 MHz) *δ* 4.82 (C × 3), 6.83 (C × 3), 9.54, 13.00, 13.14, 19.35, 24.40, 24.45, 38.39, 42.73, 65.44, 77.42, 127.40 (C × 2), 128.49, 130.70, 131.03, 131.20, 134.45, 136.50, 136.85 (C × 2), 137.62, 137.82, 141.53, 148.85, 194.41; HRMS (ESI) *m*/*z* calcd for C_31_H_48_O_3_NaSi (M + Na)^+^ 519.3265, found 519.3257.

13*Z*-Isomer: UV-VIS λ 295, 410; IR ν 3598, 3521 (OH), 1660 (conj. CO), 1612, 1598, 1565 (C=C); ^1^H-NMR (300 MHz) *δ* 0.63 (6H, q,* J* 8, SiC*H*_2_ × 3), 0.94, 0.99 (each 3H, s, *gem*-Me), 0.99 (9H, t, *J* 8, CH_2_*Me* × 3), 1.66–1.69 (5H, overlapped, 5-Me, 2-Hα, 2-Hβ), 1.88, 1.95, 2.05 (each 3H, s, 9-Me, 13-Me, 13'-Me), 4.26 (1H, m, 3-H), 5.53 (1H, s, 4-H), 5.69 (1H, d, *J* 16, 7-H), 6.16 (1H, d, *J* 12, 14-H), 6.27 (1H, d, *J* 11, 10-H), 6.47 (1H, dd, *J* 16, 8-H), 6.62 (1H, dd, *J* 11.5, 14, 15'-H), 6.75 (1H, dd, *J* 11, 15, 11-H), 6.90 (1H, d, *J* 15, 12-H), 6.97 (1H, d, *J* 11.5, 14'-H), 7.03 (1H, dd, *J* 12, 14, 15-H), 9.45 (1H, s, CHO); ^13^C-NMR (75 MHz) *δ* 4.82, (C × 3) 6.84 (C × 3), 9.52, 13.20, 19.37, 20.92, 24.41, 24.47, 38.42, 42.73, 65.45, 77.42, 126.60, 128.38, 128.55, 128.57, 129.36, 130.96, 131.09, 134.39, 136.36, 136.91, 137.10, 137.80, 140.22, 148.96, 194.49; HRMS (ESI) *m*/*z* calcd for C_31_H_48_O_3_NaSi (M + Na)^+^ 519.3265, found 519.3255.

Isomerization of 13*Z*-isomer of compound **4a**. A solution (2 mL) prepared from PdCl_2_(MeCN)_2_ (13 mg), Et_3_N (7 mL) and water (1.2 mL) in MeCN (8.8 mL) was added to a solution of 13*Z*-isomer of compound **4a** (103 mg) in MeCN (18 mL) and the mixture was stirred at rt for 3 h. The solvent was evaporated off to give a residue, which was purified by the same method described above to provide the all-*E*-isomer **4a** (54 mg, 52%).

(2*E*,4*E*,6*E*,8*E*,10*E*,12*E*)-13-[(1*R*,4*S*)-1,4-Dihydroxy-2,6,6-trimethylcyclohex-2-en-1-yl]-2,7,11-trimethyltrideca-2,4,6,8,10,12-hexaenal (**5a**). To a stirred solution of TES ether **4a** (434 mg, 0.86 mmol) in dry THF (9 mL) was added AcOH (1 M in THF; 0.30 mL, 0.30 mmol) and then TBAF (1 M in THF; 1.31 mL, 1.31 mmol) at rt. After being stirred at rt for 5 min, the mixture was evaporated to afford a residue, which was purified by flash CC (acetone-hexane, 35:65) to afford compound **5a** (280 mg, 84%) as orange solids: UV-VIS λ 421; IR ν 3603, 3446 (OH), 1660 (conj. CO), 1611, 1601, 1550 (C=C); ^1^H-NMR (500 MHz) *δ* 0.94, 1.02 (each 3H, s, *gem*-Me), 1.53, 1.62 (each 1H, br s, OH × 2), 1.67 (1H, dd, *J* 7.5, 13.5, 2-H), 1.68 (3H, t, *J* 2, 5-Me), 1.81 (1H, dd, *J* 6.5, 13.5, 2-H), 1.89 (3H, s, 13'-Me), 1.94 (3H, s, 9-Me), 2.04 (3H, s, 13-Me), 4.25 (1H, m, 3-H), 5.64 (1H, br s, 4-H), 5.69 (1H, d, *J* 15.5, 7-H), 6.24 (1H, br d, *J* 11.5, 10-H), 6.31 (1H, br d, *J* 12, 14-H), 6.39 (1H, d, *J* 15, 12-H), 6.42 (1H, d, *J* 15.5, 8-H), 6.70 (1H, dd, *J* 11.5, 14.5, 15'-H), 6.76 (1H, dd, *J* 11.5, 15, 11-H), 6.96 (1H, br d, *J* 11.5, 14'-H), 7.02 (1H, dd, *J* 12, 14.5, 15-H), 9.45 (1H, s, CHO); ^13^C-NMR (125 MHz) *δ* 9.59 (13'-Me), 13.03 (13-Me), 13.22 (9-Me), 19.21 (5-Me), 24.37 (1-Me), 24.40 (1-Me), 38.19 (C1), 42.44 (C2), 65.24 (C3), 77.83 (C6), 127.29 (C11), 127.35 (C4), 127.55 (C15'), 130.74 (C7), 131.19 (C14), 131.50 (C10), 134.44 (C8), 136.31 (C9), 136.99 (C13'), 137.10 (C12), 137.59 (C15), 138.85 (C5), 141.48 (C13), 148.83 (C14'), 194.47 (CHO); HRMS (ESI) *m*/*z* calcd for C_25_H_34_O_3_NaSi (M + Na)^+^ 405.2400, found 405.2393.

(3*R*,3'*S*,6'*R*)-Gobiusxanthin (**1a**). NaOMe (1 M in MeOH; 0.45 mL, 0.45 mmol) was added to a solution of the phosphonium salt **7** [6] (192 mg, 0.34 mmol) and the apocarotenal **5a** (86 mg, 0.23 mmol) in CH_2_Cl_2_ (10 mL) at rt. After being stirred at rt for 10 min, the mixture was poured into saturated aq. NH_4_Cl and extracted with AcOEt. The extracts were washed with brine, dried and evaporated to give a residue, which was purified by flash CC (acetone-hexane, 3:7) to provide the crude condensed products. This was dissolved in dry THF (9 mL) and then AcOH (1 M in THF; 0.30 mL, 0.30 mmol) and TBAF (1 M in THF; 0.42 mL, 0.42 mmol) were added to it at rt. After being stirred at rt for 30 min, the mixture was concentrated. The resulting residue was purified by flash CC (AcOEt-CH_2_Cl_2_-MeOH, 25:72:3) and then preparative HPLC [LiChrosorb Si 60 (7 μm) 2 × 25 cm; AcOEt-CH_2_Cl_2_-MeOH, 10:40:1] to give (3*R*,3'*S*,6'*R*)-gobiusxanthin (**1a**) (90 mg, 69% from **5a**) as orange solids. ^1^H-NMR spectral data of this synthetic **1a** were identical with those reported [1]: UV-VIS λ 277, 424, 447, 477; CD (9.83 × 10^−5^ mol/L, EPA) λ(∆ε) 211 (−5.7), 228 (−1.0), 244 (−2.7), 277 (−4.2), 304 (0), 333 (+0.5), 351 (0); IR ν 3604, 3448 (OH), 2172 (C≡C), 1568 (C=C); ^1^H-NMR (500 MHz); *δ* 0.94 (3H, s, 1'-Meα), 1.02 (3H, s, 1'-Meβ), 1.15 (3H, s, 1-Meα), 1.20 (3H, s, 1-Meβ), 1.45 (1H, t, *J* 12, 2-Hβ), 1.50, 1.61. 1.63 (each 1H, br s, OH × 3), 1.67 (1H, dd, *J* 7, 13.5, 2'-H), 1.67 (3H, t, *J* 1.5, 5'-Me), 1.81 (1H, dd, *J* 6, 13.5, 2'-H), 1.83 (1H, ddd, *J* 2, 3.5, 12, 2-Hα), 1.92 (6H, s, 5-Me, 9'-Me), 1.95, 1.97 (each 3H, s, 13-Me, 13'-Me), 2.01 (3H, s, 9-Me), 2.07 (1H, ddd, *J* 1.5, 9.5, 18, 4-Hβ), 2.43 (1H, ddd, *J* 1.5, 5.5, 18, 4-Hα), 3.99 (1H, m, 3-H), 4.24 (1H, m, 3'-H), 5.63 (1H, d, *J* 15.5, 7'-H), 5.63 (1H, m, 4'-H), 6.22 (1H, br d *J* 11.5, 10'-H), 6.27 (2H, br d, *J* 9, 14-H, 14'H), 6.35 (1H, d, *J* 14, 12-H), 6.37 (1H, d, *J* 15, 12'-H), 6.39 (1H, d, *J* 15.5, 8'-H), 6.45 (1H, dd-like, *J* 1, 11.5, 10-H), 6.51 (1H, dd, *J* 11.5, 14, 11-H), 6.62 (1H, dd, *J* 11.5, 15, 11'-H), 6.64 (2H, m, 15-H, 15'-H); ^13^C-NMR (125 MHz) *δ* 12.73, 12.81 (13-M, 13'-Me), 13.13 (9'-Me), 18.04 (9-Me), 19.22 (5'-Me), 22.47 (5-Me), 24.37 (1'-Me), 24.40 (1'-Me), 28.76 (1-Me), 30.50 (1-Me), 36.61 (C1), 38.16 (C1'), 41.46 (C4), 42.47 (C2'), 46.68 (C2), 64.86 (C3), 65.28 (C3'), 77.87 (C6'), 89.00 (C7), 98.62 (C8), 118.96 (C9), 124.16 (C11), 124.22 (C6), 124.84 (C11'), 127.22 (C4'), 129.67 (C7'), 130.08, 130.42 (C15, C15'), 131.98 (C10'), 132.74, 133.44 (C14, C14'), 134.53 (C9'), 134.60 (C8'), 135.17 (C10), 136.22, 136.63 (C13, C13'), 137.26 (C5), 137.98, 138.05 (C12, C12'), 139.01 (C5'); HRMS (ESI) *m*/*z* calcd for C_40_H_54_O_3_Na (M + Na)^+^ 605.3965, found 605.3962.

Ethyl (2*E*,4*E*)-5-[(1*R*,4*R*)-1-Hydroxy-2,6,6-trimethyl-4-triethylsilyloxycyclohex-2-en-1-yl]-3-methylpenta-2,4-dienoate (**2b**). To a stirred solution of *anti*-diol **8** [4] (465 mg, 1.58 mmol), Et_3_N (0.66 mL, 5.4 mmol) and *N,N*-dimethyl-4-aminopyridine (19 mg, 0.16 mmol) in dry CH_2_Cl_2_ (7 mL) was added TESCl (0.40 mL, 2.4 mmol) at 0 °C. The mixture was stirred at 0 °C for 30 min, poured into saturated aq. NH_4_Cl and extracted AcOEt. The extracts were washed with brine, dried and evaporated to give a residue, which was purified by flash CC (AcOEt- hexane, 1:4) to afford TES ether **2b** (645 mg, quant.) as a colorless oil: [α]_D_^26^ 157.89 (*c* 0.91, MeOH); IR ν 3605, 3473 (OH), 1704 (conj. CO), 1632, 1612 (C=C); ^1^H-NMR (300 MHz)* δ* 0.62 (6H, q,* J* 8, SiC*H*_2_ × 3), 0.87, 1.02 (each 3H, s, *gem*-Me), 0.98 (9H, t, *J* 8, SiCH_2_*Me* × 3), 1.28 (3H, t, *J* 7.5, OCH_2_*Me*), 1.60 (3H, m, 5-Me), 1.66 (2H, d-like, *J* 8, 2-H_2_), 2.27 (3H, br s, 9-Me), 4.17 (2H, q, *J* 7.5, OCH_2_), 4.28 (1H, m, 3-H), 5.47 (1H, br s, 4-H), 5.81 (1H, br s, 10-H), 6.14 (1H, d, *J* 16, 7-H), 6.34 (1H, d, *J* 16, 8-H); ^13^C-NMR (75 MHz) *δ* 4.82 (C × 3), 6.87 (C × 3), 14.11, 14.31, 17.53, 22.66, 25.16, 39.65, 44.53, 59.71, 65.91, 79.09, 119.38, 128.70, 132.51, 136.94, 138.15, 151.57, 167.14; HRMS (ESI) *m*/*z* calcd for C_23_H_40_O_4_Si (MH)^+^ 409.2769, found 409.2764.

In the same procedure as preparation of (3*R*,3'*S*,6'*R*)-gobiusxanthin (**1a**), (3*R*,3'*R*,6'*R*)-gobiusxanthin (**1b**) was prepared from the above (3*R*,6*R*)-dienoate **2b**.

(3*R*,6*R*)-Dienal **3b**: [α]_D_^27^ −194.8 (*c* 0.98, MeOH); IR ν 3608, 3477 (OH), 1662 (conj. CO), 1627, 1597, 1580 (C=C); ^1^H-NMR (300 MHz) *δ* 0.63 (6H, q,* J* 8, SiC*H*_2_ × 3), 0.88, 1.03 (each 3H, s, *gem*-Me), 0.97 (9H, t, *J* 8, CH_2_*Me* × 3), 1.60 (3H, t, *J* 2, 5-Me), 1.68 (2H, d-like, *J* 8, 2-H_2_), 2.25 (3H, d, *J* 1, 9-Me), 4.30 (1H, m, 3-H), 5.48 (1H, m, 4-H), 5.98 (1H, br d, *J* 8, 10-H), 6.31, 6.46 (each 1H, d, *J* 15.5, 7-H, 8-H), 10.10 (1H, *J* 8, CHO); ^13^C-NMR (75 MHz) *δ* 4.83 (C × 3), 6.83 (C × 3), 13.38, 17.48, 22.64, 25.18, 39.77, 44.62, 65.85, 79.16, 128.95, 129.65, 132.13, 136.70, 140.21, 154.02, 191.51; HRMS (ESI) *m*/*z* calcd for C_21_H_36_O_3_NaSi (M + Na)^+^ 387.2326, found 387.2319.

(3*R*,6*R*)-Hexaenel **5b**: UV-VIS λ 420; IR ν 3604, 3446 (OH), 1660 (conj. CO), 1611, 1600, 1550 (C=C); ^1^H-NMR (500 MHz) *δ* 0.92, 1.04 (each 3H, s, *gem*-Me), 1.46 (1H, br s, 3-OH), 1.53 (1H, br d, *J* 2, 6-OH), 1.58 (1H, dd, *J* 10, 13.5, 2-H), 1.66 (3H, t, *J* 2, 5-Me), 1.79 (1H, ddd, *J* 1.5, 6.5, 13.5, 2-H), 1.88 (3H, d, *J* 0.5, 13'-Me), 1.94 (3H, d, *J* 0.5, 9-Me), 2.03 (3H, s, 13-Me), 4.30 (1H, m, 3-H), 5.57 (1H, quint-like, *J* 1.5, 4-H), 5.79 (1H, d, *J* 16, 7-H), 6,23 (1H, br d, *J* 11, 10-H), 6.31 (1H, br d, *J* 12, 14-H), 6.32 (1H, d, *J* 16, 8-H), 6.38 (1H, d, *J* 15, 12-H), 6.69 (1H, dd, *J* 11.5, 14.5, 15'-H), 6.75 (1H, dd, *J* 11 and 15, 11-H), 6.96 (1H, br d, *J* 11.5, 14'-H), 7.02 (1H, dd, *J* 12, 15, 15-H), 9.45 (1H, s, CHO); ^13^C-NMR (125 MHz) *δ* 9.59 (13'-Me), 13.03 (13-Me), 13.27 (9-Me), 17.89 (5-Me), 22.45, 25.05 (*gem*-Me), 39.91 (C1), 44.15 (C2), 65.80 (C3), 79.41 (C6), 127.25, 127.27 (C4, C4'), 127.58 (C15'), 131.23 (C14), 131.75 (C10), 132.15 (C7), 134.18 (C8), 136.39 (C9), 137.03 (C13'), 137.17 (C12), 137.58 (C15), 138.76 (C5), 141.48 (C13), 148.76 (C14'), 194.43 (CHO); HRMS (ESI) *m*/*z* calcd for C_25_H_34_O_3_Na (M + Na)^+^ 405.2400, found 405.2396.

(3*R*,3'*R*,6'*R*)-Gobiusxanthin (**1b**): UV-VIS λ 278, 424, 448, 478; CD (1.08 × 10^−4^ mol/L, EPA) λ(∆ε) 209 (−2.9), 219 (−1.9), 241 (−9.5), 259 (0), 279 (+4.0), 294 (0), 337 (−5.0), 369 (−0.6); IR ν 3606, 3446 (OH), 2172 (C≡C), 1568 (C=C); ^1^H-NMR (500 MHz) *δ* 0.92 (3H, s, 1'-Meα), 1.03 (H, s, 1'-Meβ), 1.14 (3H, s, 1-Meα), 1.20 (3H, s, 1-Meβ), 1.45 (1H, t, *J* 12, 2-Hβ), 1.57 (1H, dd, *J* 10 and 13, 2'-Hα), 1.66 (3H, t, *J* 1.5, 5'-Me), 1.79 (1H, ddd, *J* 1, 6, 13, 2'-Hβ), 1.84 (1H, ddd, *J* 1.5, 3.5, 12, 2-Hα), 1.92 (6H, s, 5-Me, 9'-Me), 1.95 (3H, s, 13'-Me), 1.97 (3H, s, 13-Me), 2.00 (3H, s, 9-Me), 2.07 (1H, br dd, *J* 9, 18, 4-Hβ), 2.43 (1H, ddd, *J* 1.5, 5.5, 18, 4-Hα), 3.99 (1H, m, 3-H), 4.29 (1H, m, 3'-H), 5.57 (1H, m, 4'-H), 5.73 (1H, d, *J* 15.5, 7'-H), 6.21 (1H, br d, *J* 11.5, 10'-H), 6.26, 6.27 (each 1H, br d, *J* 9, 14-H, 14'-H), 6.30 (1H, d, *J* 15.5, 8'-H), 6.35 (1H, d, *J* 14.5, 12-H), 6.36 (1H, d, *J* 15, 12'-H), 6.45 (1H, dd-like, *J* 1, 11.5, 10-H), 6.51 (1H, dd, *J* 11.5, 14.5, 11-H), 6.61 (1H, dd, *J* 11.5, 15, 11'-H), 6.64 (2H, m, 15-H, 15'-H); ^13^C-NMR (125 MHz) *δ* 12.75, 12.83 (13-Me, 13'-Me), 13.20 (9'-Me), 17.95 (5'-Me), 18.06 (9-Me), 22.48, 22.50 (5-Me, 1'-Meβ), 25.07 (1'-Meα), 28.79 (1-Meα), 30.53 (1-Meβ), 36.64 (C1), 39.93 (C1'), 41.51 (C4), 44.19 (C2'), 46.74 (C2), 64.91 (C3), 65.88 (C3'), 79.45 (C6'), 89.04 (C7), 98.66 (C8), 119.00 (C9), 124.19 (C11), 124.29 (C6), 124.86 (C11'), 127.14 (C4'), 130.13, 130.45, (C15, C15') 131.12 (C7'), 132.27 (C10'), 132.81 (C14'), 133.47 (C14), 134.43 (C8'), 134.62 (C9'), 135.20 (C10), 136.25 (C13), 136.67 (C13'), 137.26 (C5), 138.07, 138.09 (C12, C12'), 138.92 (C5'); HRMS (ESI) *m*/*z* calcd for C_40_H_55_O_3_ (M + H)^+^ 583.4146, found 583.4140.

### 3.3. Synthesis of Gobiusxanthin Stereoisomers **1c**–**1f**

Ethyl (2*E*,4*E*)-5-[(*S*)-1-Hydroxy-2,6,6-trimethyl-4-oxocyclohex-2-en-1-yl]-3-methylpenta-2,4- dienoate (**10**). To a mixture of epoxide **9** [9] (2.52 g, 8.57 mmol), NaHCO_3_ (800 mg, 9.5 mmol) in CH_2_Cl_2_ (50 mL) was added DMP (4.72 g, 11.1 mmol) in some portions at rt and the mixture was stirred for a farther 30 min. After CH_2_Cl_2_ was evaporated off, the resulting mixture was diluted with eher-hexane (1:1) and filtered through a pad of Celite. The filtrate was evaporated to afford a residue, which was purified by flash CC (AcOEt-hexane, 2:3) to afford the crude epoxy ketone (2.53 g), which was dissolved in AcOEt (400 mL) and silica gel (70–230 mesh Merck-1.07734; 130 g) was added to it. After being stirred at rt for 18 h, the mixture was filtered through sintered glass funnel and the filtrate was evaporated. The residue was purified by flash CC (AcOEt-hexane, 35:65) to provide (6*S*)-enone **10** (2.05 g, 82% from **9**) as colorless solids: Its spectral data were identical with those of previously reported [4] (6*R*)-enone: [α]_D_^22^ 340.8 (*c* 0.97, MeOH); HRMS (ESI) *m*/*z* calcd for C_17_H_24_O_4_Na (M + Na)^+^ 315.1567, found 315.1563.

Reduction of enone **10** with 9-BBN. To a stirred solution of enone **10** (2.00 g, 6.85 mmol) in dry THF (50 mL) was added dropwise 9-BBN (0.5 M in THF; 29 mL. 14.5 mmol) at 0 °C and the mixture was stirred at rt for 2 h. The reaction was quenched by addition of MeOH (5 mL) followed by 2-aminoethanol (1 mL) and the mixture was stirred at rt for 15 min. The mixture was concentrated and the resulting residue was purified by flash CC (acetone-hexane, 3:7) and then preparative HPLC (COSMOSIL 5SL-II 2 × 25 cm; MeOH-CH_2_Cl_2_, 2.5:100) to afford (3*R*,6*S*)-*syn*-diol **11a** (950 mg, 47%) and (3*S*,6*S*)-*anti*-diol **11b** (890 mg, 44%) as a colorless viscous oil, respectively. Their spectral data except for optical data were identical with those of previously reported [4] (3*S*,6*R*)-*syn*-diol and (3*R*,6*R*)-*anti*-diol **8**.

(3*R*,6*S*)-*Syn*-diol **11a**: [α]_D_^21^ 140.1 (*c* 0.99, MeOH); HRMS (ESI) *m*/*z* calcd for C_17_H_26_O_4_Na (M + Na)^+^ 317.1723, found 317.1714.

(3*S*,6*S*)-*Anti*-diol **11b**: [α]_D_^20^ 240.9 (*c* 1.05, MeOH); HRMS (ESI) *m*/*z* calcd for C_17_H_26_O_4_Na (M + Na)^+^ 317.1723, found 317.1719.

In the same procedure as preparation of (3*R*,3'*S*,6'*R*)-gobiusxanthin (**1a**), (3*R*,3'*S*,6'*S*)-gobiusxanthin (**1c**) and (3*R*,3'*R*,6'*S*)-gobiusxanthin (**1d**) were prepared from above (3*R*,6*S*)-*syn*-diol **11a** and (3*S*,6*S*)-*anti*-diol **11b**, respectively. (3*S*,3'*S*,6'*R*)-Gobiusxanthin (**1e**) and (3*S*,3'*S*,6'*S*)-gobiusxanthin (**1f**) were prepared by using the enantiomer [11] of C_15_-phosphonium salt **7**. Spectral data except for optical data of compounds **1c–f**, **12a**,**b**, **13a**,**b** and **14a**,**b** were identical with the corresponding enantiomers **2a**,**b**, **3a**,**b**, **5a**,**b** and diastereomers **1a**,**b**.

(3*R*,6*S*)-*Syn*-dienoate **12a**: [α]_D_^18^ 118.3 (*c* 1.03, MeOH); HRMS (ESI) *m*/*z* calcd for C_23_H_40_O_4_NaSi (M + Na)^+^ 431.2588, found 431.2589.

(3*R*,6*S*)-*Syn*-dienal **13a**: [α]_D_^18^ 159.4 (*c* 1.04, MeOH); HRMS (ESI) *m*/*z* calcd for C_21_H_36_O_3_NaSi (M + Na)^+^ 387.2326, found 387.2321.

(3*R*,6*S*)-*Syn*-apocarotenal **14a**: HRMS (ESI) *m*/*z* calcd for C_25_H_34_O_3_Na (M + Na)^+^ 405.2400, found 405.2391.

(3*R*,3'*R*,6'*S*)-Gobiusxanthin (**1c**): CD (1.07 × 10^−4^ mol/L, EPA) λ(∆ε) 205 (−2.0), 209 (0), 216 (+1.5), 227 (+0.5), 246 (+2.1), 277 (+3.0), 304 (0), 325 (−0.2), 345 (0); HRMS (ESI) *m*/*z* calcd for C_40_H_54_O_3_Na (M + Na)^+^ 605.3965, found 605.3965.

(3*S*,6*S*)-*Anti*-dienoate **12b**: [α]_D_^19^ 164.4 (*c* 1.07, MeOH); HRMS (ESI) *m*/*z* calcd for C_23_H_40_O_4_NaSi (M + Na)^+^ 431.2588, found 431.2587.

(3*S*,6*S*)-*Anti*-dienal **13b**: [α]_D_^18^ 265.8 (*c* 0.97, MeOH); HRMS (ESI) *m*/*z* calcd for C_21_H_36_O_3_NaSi (M + Na)^+^ 387.2326, found 387.2321.

(3*S*,6*S*)-*Anti*-apocarotenal **14b**: HRMS (ESI) *m*/*z* calcd for C_25_H_34_O_3_Na (M + Na)^+^ 405.2400, found 405.2391.

(3*R*,3'*S*,6'*S*)-Gobiusxanthin (**1d**): CD (9.91 × 10^−5^ mol/L, EPA) λ(∆ε) 205 (−4.5), 219 (0), 242 (+8.0), 259 (0), 279 (+4.4), 294 (0), 338 (+4.9), 367 (+0.7); HRMS (ESI) *m*/*z* calcd for C_40_H_54_O_3_Na (M + Na)^+^ 605.3965, found 605.3956.

(3*S*,3'*S*,6'*R*)-Gobiusxanthin (**1e**): CD (9.68 × 10^−5^ mol/L, EPA) λ(∆ε) 208 (0), 214 (−1.4), 229 (−0.3), 244 (−1.2), 276 (−2.3), 289 (0), 330 (+0.3), 352 (0); HRMS (ESI) *m*/*z* calcd for C_40_H_54_O_3_Na (M + Na)^+^ 605.3965, found 605.3962.

(3*S*,3'*S*,6'*S*)-Gobiusxanthin (**1f**): CD (1.10 × 10^−4^ mol/L, EPA) λ(∆ε) 211 (+1.6), 218 (+1.1), 241 (+7.16), 260 (0), 279 (−3.1), 293 (0), 338 (+4.2), 369 (+0.6); HRMS (ESI) *m*/*z* calcd for C_40_H_53_O_3_ (M − H)^−^ 581.3990, found 581.4013.

### 3.4. Isolation of Epigobiusxanthin

The crown-of-thorns starfish *Acanthaster planci,* collected at the Ootsuki coast, Kochi Prefecture, Japan (10 specimens 1,870 g), was extracted with acetone. The extract was partitioned between ether-hexane (1:1) and water. The organic layer was dried over Na_2_SO_4_ and then evaporated. The residual red-colored oil was chromatographed on silica gel using an increasing percentage of acetone in hexane. The fraction eluted with acetone-hexane (6:4) was subjected to HPLC on silica gel with acetone-hexane (4:6) and then on ODS silica with CHCl_3_-MeCN (2:8) to yield epigobiusxanthin (0.10 mg).

## 4. Conclusions

In summary, we achieved the first total syntheses of gobiusxanthin (**1a**), 3'-epigobiusxanthin (**1b**) and other stereoisomers **1c**–**f** via the stereoselective Wittig reactions of the (3*R*)-C_15_-acetylenic tri-*n*-butylphosphonium salt **7** and its enantiomer with the corresponding C_25_-apocarotenal stereoisomers **5a**,**b** and **14a**,**b**. The absolute configurations of the 3',6'-dihydroxy-ε-end moieties in these two carotenoids were determined by comparison of their CD spectra with those of synthetic samples: 3'*S*,6'*R* and 3'*S*,6'*S* configurations were deduced for gobiusxanthin and epigobiusxanthin, respectively. In addition, a HPLC separation method for (3*R*)- and (3*S*)-stereoisomers was established by using a chiral column. The HPLC analysis has proved that the stereochemistry of the natural epigobiusxanthin is 3*R*,3'*S*,6'*S*: natural epigobiusxanthin is 6'-epi-isomer rather than 3'-epi-isomer of gobiusxanthin.

The present research has indicated that HPLC analysis can be a strong tool to determine the absolute stereochemistries of chiral compounds, especially those having multiple chirogenic centers. To do this, in a concerted manner, development of a total synthetic method is essential to supply a sterically-defined authentic sample.
